# Demographic risk assessment for a harvested species threatened by climate change: polar bears in the Chukchi Sea

**DOI:** 10.1002/eap.2461

**Published:** 2021-10-26

**Authors:** Eric V. Regehr, Michael C. Runge, Andrew Von Duyke, Ryan R. Wilson, Lori Polasek, Karyn D. Rode, Nathan J. Hostetter, Sarah J. Converse

**Affiliations:** ^1^ Polar Science Center Applied Physics Laboratory University of Washington Seattle Washington 98105 USA; ^2^ Patuxent Wildlife Research Center U.S. Geological Survey Laurel Maryland 20708 USA; ^3^ Department of Wildlife Management North Slope Borough Utqiaġvik Alaska 99723 USA; ^4^ Marine Mammals Management U.S. Fish and Wildlife Service Anchorage Alaska 99503 USA; ^5^ Division of Wildlife Conservation Alaska Department of Fish and Game Juneau Alaska 99802 USA; ^6^ Alaska Science Center U.S. Geological Survey Anchorage Alaska 99508 USA; ^7^ Washington Cooperative Fish and Wildlife Research Unit School of Aquatic and Fishery Sciences University of Washington Seattle Washington 98105 USA; ^8^ Washington Cooperative Fish and Wildlife Research Unit School of Environmental and Forest Sciences (SEFS) & School of Aquatic and Fishery Sciences (SAFS) U.S. Geological Survey University of Washington Seattle Washington 98105 USA

**Keywords:** climate change, density dependence, habitat loss, harvest, polar bear, risk assessment, state‐dependent management, subsistence, sustainability, *Ursus maritimus*

## Abstract

Climate change threatens global biodiversity. Many species vulnerable to climate change are important to humans for nutritional, cultural, and economic reasons. Polar bears *Ursus maritimus* are threatened by sea‐ice loss and represent a subsistence resource for Indigenous people. We applied a novel population modeling‐management framework that is based on species life history and accounts for habitat loss to evaluate subsistence harvest for the Chukchi Sea (CS) polar bear subpopulation. Harvest strategies followed a state‐dependent approach under which new data were used to update the harvest on a predetermined management interval. We found that a harvest strategy with a starting total harvest rate of 2.7% (˜85 bears/yr at current abundance), a 2:1 male‐to‐female ratio, and a 10‐yr management interval would likely maintain subpopulation abundance above maximum net productivity level for the next 35 yr (approximately three polar bear generations), our primary criterion for sustainability. Plausible bounds on starting total harvest rate were 1.7–3.9%, where the range reflects uncertainty due to sampling variation, environmental variation, model selection, and differing levels of risk tolerance. The risk of undesired demographic outcomes (e.g., overharvest) was positively related to harvest rate, management interval, and projected declines in environmental carrying capacity; and negatively related to precision in population data. Results reflect several lines of evidence that the CS subpopulation has been productive in recent years, although it is uncertain how long this will last as sea‐ice loss continues. Our methods provide a template for balancing trade‐offs among protection, use, research investment, and other factors. Demographic risk assessment and state‐dependent management will become increasingly important for harvested species, like polar bears, that exhibit spatiotemporal variation in their response to climate change.

## Introduction

Climate change is projected to result in loss of species (Bellard et al. 2012), with far‐ranging consequences for humans, including effects on commercial and subsistence food sources. In recent decades, the Arctic has warmed faster than other regions, resulting in melting of glaciers and sea ice (Post et al. 2019). Arctic marine mammals that depend on ice and have specialized feeding requirements are particularly vulnerable to such changes (Laidre et al. 2008). Because >75% of Arctic marine mammal stocks are regularly and legally harvested for subsistence (Laidre et al. 2015), climate change presents new challenges for hunters, communities, and managers seeking to balance population protection with continued opportunities for use.

Polar bears occur throughout the circumpolar Arctic in 19 subpopulations as recognized by the International Union for the Conservation of Nature Polar Bear Specialist Group (Durner et al. 2018). They depend on ice to access their primary prey, ringed seals *Pusa hispida* and bearded seals *Erignathus barbatus*. In 2008, polar bears were listed as “threatened” under the U.S. Endangered Species Act due to sea‐ice loss resulting from climate change (USFWS 2016). Although most polar bears will likely experience negative effects in the long term (Atwood et al. 2016, Regehr et al. 2016), the status of the 19 subpopulations currently varies due to differences in physical geography, biological productivity, sea‐ice dynamics, and other factors (Durner et al. 2018).

The Chukchi Sea (CS) polar bear subpopulation ranges across the northern Bering, Chukchi, and East Siberian seas (Wilson et al. 2016). Management authority occurs under an agreement between the United States and Russia (United States T. Doc. 107‐10) that provides for legal subsistence harvest and requires identification of an annual sustainable harvest level based on scientific data and Indigenous Knowledge (IK). Under this agreement, management decisions are made by a four‐member commission with governmental and Indigenous representation from each country. Recent research suggests that the body condition (i.e., fatness), reproduction, and demographic status of the CS subpopulation have been positive in recent years (Rode et al. 2014, 2021, Regehr et al. 2018), which is consistent with IK assessments (Voorhees et al. 2014, Braund et al. 2018). However, climate change is affecting the ecology and habitat use of CS bears (Rode et al. 2018). Between 1986 and 2013, the percent of females summering on land increased from 20% to 39% and the average time spent on land increased by 30 d (Rode et al. 2015). Energetics models linking the duration of fasting on land to recruitment and survival suggest that reduced on‐ice hunting opportunities could lead to demographic declines for CS bears by ˜2040 (Molnár et al. 2020). Similar declines have already been observed for some other polar bear subpopulations (Lunn et al. 2016).

Our objective was to evaluate the demographic effects of harvest as a function of environmental conditions, population vital rates (e.g., reproduction and survival), and practical aspects of harvest that managers can control, including the harvest rate and sex ratio, management interval, and level of precision in population data. In this paper, “harvest” refers to all human‐caused removals (i.e., the combination of subsistence harvest, bears killed in defense of life and property, and other sources of direct human‐caused mortality). With estimates of vital rates and abundance from an integrated population model for CS bears (CS‐IPM; Regehr et al. 2018), we used a stage‐based matrix model to perform stochastic population projections that account for harvest, habitat loss, density dependence, and the design of future research and monitoring studies to inform harvest management. We evaluated harvest effects over approximately three polar bear generations (35 yr; Regehr et al. 2016) under alternative assumptions for future trends in environmental carrying capacity. During population projections, harvest levels were updated periodically using new estimates of vital rates and abundance under a state‐dependent (i.e., dependent on current conditions) management framework (Runge et al. 2009). Results consist of a range of potential harvest strategies and their forecasted demographic outcomes.

## Materials and Methods

Our assessment applies to the CS subpopulation area, with the southern boundary modified to exclude regions not used by bears during 2008–2016 (Regehr et al. 2018). Chukchi Sea bears live on the sea ice from fall through spring. In summer, some individuals retreat north with the melting ice while others come onto land, especially Russia’s Wrangel Island (Rode et al. 2015).

**Fig. 1:**
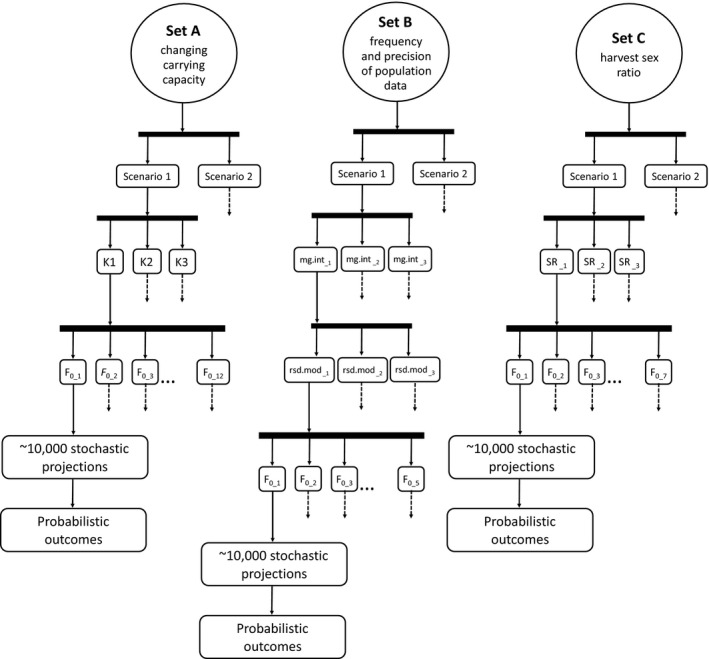
Simulation approach to evaluate harvest risk for the Chukchi Sea polar bear subpopulation. We evaluated three sets of simulations that investigated different aspects of harvest management: (A) assumptions for future carrying capacity (*K*1, *K*2, and *K*3), (B) management interval (*mg.int*) and precision of future population data (*rsd.mod*), and (C) harvest sex ratio (SR). All sets of simulations evaluated two scenarios of vital rates (scenario 1 and scenario 2) and multiple levels of the management factor that adjusted the harvest rate (*F*_0_). Each combination of settings was evaluated using ˜10,000 stochastic projections of demographic and harvest processes. See *Methods* for detailed descriptions.

We developed three sets of simulations to investigate factors relevant to harvest management (Fig. 1). Set A evaluated alternative assumptions for future carrying capacity. Set B evaluated the management interval and precision of population data. Set C evaluated the harvest sex ratio. All sets included two scenarios of vital rates corresponding to lower (scenario 1) and higher (scenario 2) population growth rates, reflecting uncertainty in the demographic status of CS bears. For each scenario, we considered a range of harvest rates by specifying different values of a management factor. Below, we describe the matrix projection model, the state‐dependent harvest management framework, and simulations using the matrix model to project harvested subpopulations forward while recording demographic outcomes at each time step. Details are provided in the supporting information.

Computations were performed in the R computing language (version R 3.4.0; R Core Team 2021) using the package popbio (Stubben and Milligan 2007). Unless otherwise noted, we report median values.

### Matrix projection model

**Fig. 2:**
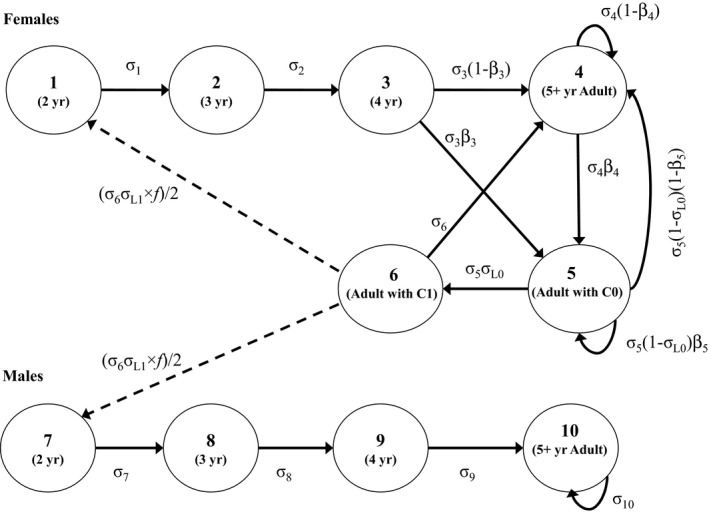
The polar bear life cycle graph underlying the matrix‐based projection model. Stages 1–6 are females and stages 7–10 are males; $\sigma_{i}$ is the annual probability of survival of an individual in stage *i*; $\sigma_{L0}$ and $\sigma_{L1}$ are the probabilities of at least one member of a cub‐of‐the‐year (C0) or yearling (C1) litter surviving; *f* is the expected size of C1 litters that survive to 2 yr; and $\beta_{i}$ is the probability, conditional on survival, of an individual in stage *i* breeding, thereby producing a C0 litter with at least one member surviving. Solid lines are stage transitions and dashed lines are reproductive contributions. Although we do not include notation for time dependence (*t*) for simplicity, all vital rates could vary during population projections due to density dependence. The life cycle graph was reproduced from Fig. 1 in Regehr et al. (2017).

The matrix projection model was based on the polar bear life cycle (Fig. 2; Regehr et al. 2017). Transitions among stages were defined by vital rates relative to a post‐breeding census from the spring of year *t* to the spring of year *t* + 1. Projections were referenced to independent bears ≥2 yr old because dependent young (cubs of the year [C0s] and yearlings [C1s]) were not included as individuals in the life cycle, but rather were used to define the reproductive status of their mother. Following Regehr et al. (2017), we did not allow a female with C1s (stage 6) to transition directly to a female with C0s (stage 5) because the estimated probability of this transition was negligible (Regehr et al. 2018). To incorporate negative density dependence, each vital rate declined with relative density (i.e., the ratio of abundance [*N*] to carrying capacity [*K*]) at a rate inversely proportional to its elasticity, according to density‐dependent curves developed previously for polar bears (Regehr et al. 2017). To incorporate positive density dependence, reproduction declined at low densities or skewed sex ratios using a mechanistic submodel for Allee effects in the mating system (Molnár et al. 2014; Appendix S1). When the matrix projection model was populated with a set of vital rates, the corresponding intrinsic growth rate (*r*) was calculated as the log of λ, the dominant eigenvalue (Caswell 2001). Harvest calculations used the corresponding finite growth rate (*R*) defined as λ − l (Runge et al. 2009).

### State‐dependent harvest management

During population projections, harvest at each time step was calculated as follows:

(1) $$H^{\text{female}}\left(t\right)=F_{O}\times {\tilde{R}}_{\text{MNPL}}\left(t\right)\times \tilde{N}\left(t\right)\times 0.5$$

and

(2) $$H^{\text{male}}\left(t\right)=H^{\text{female}}\left(t\right)\times \text{SR}$$

where $H^{\text{female}}\left(t\right)$, is the female harvest level, defined as the number of females that can be removed in year *t*, *F*_0_ is a user‐specified management factor that adjusts the harvest rate to reflect management objectives and risk tolerance, ${\tilde{R}}_{\text{MNPL}}\left(t\right)$ is the median estimate of finite growth rate in year *t* from a research study, referenced to a relative density corresponding to maximum net productivity level (MNPL), the subpopulation size that results in the greatest net annual increment in numbers resulting from reproduction minus losses due to natural mortality, $\tilde{N}\left(t\right)$ is an estimate of abundance in year *t* from a research study, selected as the 15th percentile of its sampling distribution to protect against overharvest when sampling uncertainty is large (i.e., like the minimum population estimate used in the Potential Biological Removal approach [Wade 1998]), 0.5 is a factor to calculate female removals assuming an equal subpopulation sex ratio, which protects against excessive female removals when the male segment of a subpopulation is depleted, $H^{\text{male}}$(*t*) is the male harvest level, and SR is a factor that specifies the male‐to‐female ratio in removals. For context, we note that user‐specified values of *F*_0_ = 1 and SR = 1 would result in a total harvest level (i.e., $H^{\text{female}}+H^{\text{male}}$) equal to the theoretical maximum sustainable yield, provided that subpopulation size was equal to MNPL, the parameters ${\tilde{R}}_{\text{MNPL}}$ and $\tilde{N}$ were set at the true values of $R_{\text{MNPL}}$ and N, and the subpopulation consisted of 50% females.

Using Eqs. 1 and 2, the harvest rate at each time step, referenced to the 15th percentile of the sampling distribution for abundance of independent bears, is calculated as $h\left(t\right)=\left[H^{\text{female}}\left(t\right)+H^{\text{male}}\left(t\right)\right]/\tilde{N}\left(t\right)$. During simulations, we imposed an upper limit of 0.10 on h to mitigate demographic risk associated with large uncertainty in estimates of $r_{\text{MNPL}}$ from the CS‐IPM (see *Results*). This corresponded to an upper limit of ˜0.05 on the total harvest rate, $h^{\text{total}}$, defined as the percentage of the 50th percentile of total abundance (i.e., the number of independent bears and dependent young of both sexes) removed annually. We evaluated the effects of this constraint by performing supplemental simulations with a higher upper limit on $h^{\text{total}}$ (Appendix S2).

We explored a series of harvest strategies defined by unique combinations of *F*_0_, SR, management interval (*mg.int*), and uncertainty (*rsd.mod*). The *mg.int* specified the number of years between changes to the harvest based on new population data. During population projections, $\tilde{N}\left(t=1\right)$ and ${\tilde{R}}_{\text{MNPL}}\left(t=1\right)$ were obtained from the CS‐IPM. On subsequent time steps, we simulated research studies that provided new data to inform harvest management by selecting values of ${\tilde{R}}_{\text{MNPL}}\left(t\right)$ and $\tilde{N}\left(t\right)$ from a multivariate normal distribution, with means corresponding to true values in the projection and a covariance structure calculated from the CS‐IPM. This approach assumed stationary statistical relationships among parameters. Uncertainty in simulated research studies was calculated as the product of a modifier (*rsd.mod*) and the relative standard deviation (rsd) of estimates of $R_{\text{MNPL}}$ and *N* from the CS‐IPM (see *Vital rate scenarios*).

### Simulations

Sets A, B, and C of simulations included 72, 90, and 42 simulations, respectively. Each simulation consisted of ˜10,000 stochastic projections using the same starting vital rates (see *Vital rate scenarios*), mean environmental conditions, and harvest strategy. We considered total harvest rates ranging from ˜0% to 5% by specifying different values of *F*_0_ (range 0–2) and SR (range 1–2) in Eqs. 1 and 2. Each set of simulations focused on a different aspect of harvest management by keeping some inputs constant while varying others (Fig. 1).

Operations during population projections are described in Appendix S3. In brief, at each time step, (1) the subpopulation was projected forward one year using the matrix model; (2) harvest was calculated using Eqs. 1 and 2, and allocated across life cycle stages based on the proportional stage distribution and estimates of stage‐specific harvest vulnerability; (3) relative density was calculated as N/K, expressed as metabolic energetic equivalents; and (4) density effects were imposed through the density‐dependent curves of the vital rates and the submodel for Allee effects. Every several years, as specified by *mg.int*, simulated research studies provided new values of ${\tilde{R}}_{\text{MNPL}}\left(t\right)$ and $\tilde{N}\left(t\right)$ that were used to calculate harvest until the next management interval. To incorporate sampling uncertainty, the matrix model was parameterized at *t* = 1 with estimates of vital rates (corresponding to either scenario 1 or scenario 2; see *Vital rate scenarios*) and abundance from one iteration of the Markov chain Monte Carlo results from the CS‐IPM. To incorporate environmental variation, five projections were performed for each vital rate sample while allowing for stochastic variation in projected *K* (Appendix S4).

#### Set A: changing carrying capacity

Although sea‐ice loss due to climate change is expected to negatively impact polar bears throughout their range (Atwood et al. 2016), it is not possible to accurately forecast near‐term trends in the demographic status of the CS subpopulation based on existing information. Therefore, in set A of simulations, we evaluated harvest under three plausible assumptions for future changes in *K* resulting from habitat loss. At *t* = 1, *K* was calculated from the starting value of *N* and a user‐specified value of relative density (see *Vital rate scenarios*). At *t* = 2, 3, … 36, the trend and variation in *K* were based on a stochastic projection of the standardized number of ice‐covered days per year within the CS subpopulation boundary (Stern and Laidre 2016; Appendix S4), under one of three methods:

*K*1: Trend and variation in *K* were projected forward using a linear model fit to the number of ice‐covered days during 1979–2016. This resulted in a relatively gradual decline (slope = −1.04 ice‐covered days/yr, standard error of the slope = 0.20, *P* < 0.001, root mean squared error [RMSE] = 13.5).
*K*2: The linear model was fit to the number of ice‐covered days during 2000–2016, resulting in faster declines (slope = −1.57 ice‐covered d/yr, standard error of the slope = 0.70, *P* = 0.04, RMSE = 14.0) and more interannual variation. *K*2 represents the assumption that future sea‐ice declines will occur at the rate observed since a physical regime shift in the CS region in the year 2000 (Frey et al. 2015).
*K*3: The linear model had slope = 0 for *t* = 1, 2, … 17, and the slope from *K*2 for *t* = 18, 19, … 36. Based on our interpretation of the biological effects of forecasted sea‐ice changes from global climate models and IK (Appendix S4), *K*3 represents the assumption that *K* will remain stable until 2036 and then decline.

For set A of simulations, only *F*_0_ and the method to project *K* varied. Management interval (*mg.int*) and data precision (*rsd.mod*) were fixed to 10 yr and 1.0, respectively. The harvest sex ratio (SR) was 2.0 (Fig. 1).

#### Set B: frequency and precision of new population data

Set B evaluated the effects of management update frequency and data precision. We considered three management intervals (*mg.int* = 5, 10, and 15 yr) and three precision levels (*rsd.mod* = 0.25, 0.50, and 1.0). For example, *rsd.mod* = 0.50 corresponded to future estimates of demographic parameters with relative standard deviations half as large as the CS‐IPM. For set B, carrying capacity followed *K*1 and SR was fixed to 2.0 (Fig. 1).

#### Set C: harvest sex ratio

Set C evaluated different harvest sex ratios. We considered three values for the male‐to‐female ratio in removals (SR = 1.0, 1.5, and 2.0). Carrying capacity followed *K*1, while *mg.int* and *rsd.mod* were fixed to 10 yr and 1.0, respectively (Fig. 1).

### Vital rate scenarios

Estimates of vital rates and abundance were available as 3,000 samples from posterior distributions for parameters in the CS‐IPM (Regehr et al. 2018). We used estimates of total survival (i.e., the probability of remaining alive, considering all sources of mortality, and not permanently emigrating from the study area) together with harvest data to estimate unharvested survival probability (Appendix S5). Each set of simulations was run under two vital rate scenarios (Appendix S6). Scenario 1 vital rates came directly from the CS‐IPM. Scenario 2 vital rates came from the CS‐IPM, with survival of independent bears adjusted upward to produce a mean asymptotic intrinsic growth rate of *r* = 0.05. We included scenario 2 because survival estimates for independent bears from the CS‐IPM may have included negative bias due to un‐modeled heterogeneity in recapture and movement probabilities (Regehr et al. 2018). For both scenarios, we removed biologically implausible samples corresponding to a maximum intrinsic growth rate (*r*_max_) <0 or >0.10. This resulted in removing 29% and 28% of samples from scenarios 1 and 2, respectively (Appendix S6).

The current relative density of the CS subpopulation is unknown. Therefore, under scenario 1, for each projection relative density at *t* = 1 was randomly selected from the distribution $\text{Unif}\left(0.50,0.94\right)$. The upper limit of 0.94 was based on exploratory simulations using a theta‐logistic population model (USFWS 2016) with a harvest rate of 2% and an assumed maximum growth rate *r*_max_ = 0.10 (Appendix S6). The lower limit of 0.5 corresponds to MNPL under a generalized logistic model for density dependence, which likely represents a minimum value for long‐lived species (Wade 1998). We referenced scenario 2 to a relative density corresponding to MNPL, because $r_{\text{MNPL}}$ = 0.05 is a mean estimate from case studies for 10 polar bear subpopulations (Regehr et al. 2017). Thus, scenario 2 represented the assumption that survival estimates from the CS‐IPM were negatively biased and, in fact, from 2008–2016 the CS subpopulation was capable of growth typical of other polar bear subpopulations (see *Discussion*).

### Management objectives

Our primary criterion for sustainability was to maintain subpopulation abundance above MNPL, which protects against overharvest while allowing the possibility for harvest levels to approach maximum sustainable yield (USFWS 2016). To establish a consistent reference, we calculated MNPL using N/K = 0.70 (Regehr et al. 2017). For a given simulation, the probability of meeting this management objective at the final time step (*P*_MO_) was estimated as the proportion of stochastic projections for which $N\left(t=36\right)>0.70K$. We also recorded median annual harvest at time steps *t* = 1, 18, and 36 ($H\left[t=11836\right]$); median long‐term yield, defined as the 50th percentile of the average annual harvest over the 35‐yr duration of projections (${\bar{H}}_{\text{yield}}$); probability of functional extirpation ($P_{\text{ext}}$), defined as *N* < a quasi‐extinction threshold of 100 independent bears; and probability of male depletion ($P_{\text{dep}}$) due to sex‐selective harvest, defined as <50 males in life cycle stage 10.

We report results for the subset of harvest strategies corresponding to three predetermined degrees of risk tolerance. We defined low, medium, and high risk tolerances as accepting a 10%, 30%, or 50% probability of failing to meet the management objective, respectively. For example, a harvest strategy with $P_{\text{MO}}$ = 0.90 would have a 10% probability (i.e., 1 − $P_{\text{MO}}$ = 0.10) of failing to meet the management objective, and thus would be considered sustainable at low risk tolerance. These levels of risk tolerances are used to summarize results and are not intended to represent the perspectives of an individual or organization. When a simulation set included harvest strategies that were incrementally higher and lower than would meet the management objective at a predetermined degree of risk tolerance, we used linear interpolation to approximate the specific strategy that would meet the objective.

## Results

### Vital rates and abundance

The mode of total estimated abundance from Regehr et al. (2018) was 2,937 (median = 3,194; 95% credible interval [CRI] = 1,552–5,944) during 2008–2016. The observed growth rate, estimated using vital rates directly from the CS‐IPM and thus including harvest mortality, was −0.01 (95% CRI = −0.04–0.03). The observed total harvest rate was *h* ^total^ = 2.0% (95% CRI = 1.0–4.4%; Appendix S5). These results provide context for the demographic status of CS bears.

During population projections, abundance at *t* = 1 was drawn from a distribution of 2,114 (95% CRI = 1,023–3,962) independent bears, calculated as the product of the distribution of total abundance and the stable‐stage proportion of independent bears (0.66, 95% CRI = 0.60–0.73). Under vital rate scenario 1 (Appendix S6: Table S1), intrinsic growth rate referenced to a relative density at MNPL was $r_{\text{MNPL}}$ = 0.02 (95% CRI = 0.00–0.06). Density dependence functioned like Regehr et al. (2017), with MNPL occurring at N/K = 0.73 (95% CRI = 0.69–0.74) and a ratio of subpopulation growth rate at MNPL to maximum intrinsic growth rate (i.e., *r*_MNPL_/*r*_max_) of 0.84 (95% CRI = 0.82–0.84). Under vital rate scenario 2 (Appendix S6: Table S1), survival was adjusted upward to account for potential negative bias, leading to a higher intrinsic growth rate *r*_MNPL_ = 0.04 (95% CRI = 0.00–0.07). MNPL occurred at *N*/*K* = 0.70 (95% CRI = 0.68–0.74) and the ratio *r*_MNPL_/*r*_max_ was 0.83 (95% CRI = 0.82–0.84).

#### Set A: changing carrying capacity

**Fig. 3:**
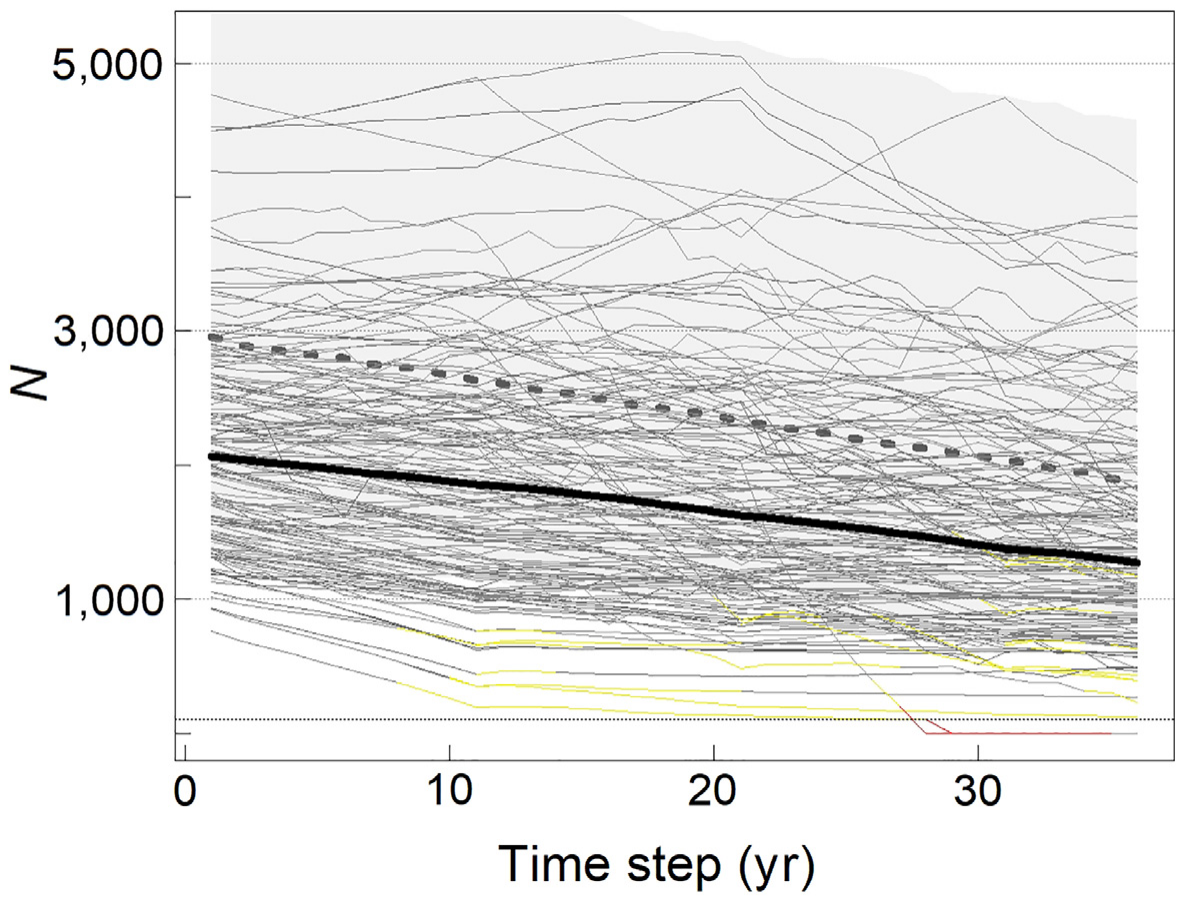
Stochastic projections (150 randomly selected iterations; thin black lines) for Chukchi Sea polar bears using vital rate scenario 1 (lower population growth rate). The heavy dashed line and shaded area represent the median and 95% confidence interval for projected carrying capacity under assumption *K*2. The *y*‐axis is subpopulation size (*N*) referenced to independent bears and the heavy black line is median subpopulation size. Individual projections are colored yellow and red for time steps at which they experienced male depletion or extirpation, respectively. The quasi‐extinction threshold is denoted by the horizontal dotted line at *N* = 100 bears. Projections are for a harvest strategy with *F*_0_ = 1.9, a 10‐yr management interval, a 2:1 male‐to‐female harvest ratio, and the baseline level of precision in population data. This equates to a starting harvest level of 82 bears/yr, which is slightly lower than the high‐risk tolerance strategy identified for scenario 1 and assumption *K*2 in Table 1. Parameters are defined in *Materials and Methods*.

A declining K required reductions in the harvest level to limit the risk of undesired demographic outcomes. Under vital rate scenario 1 (lower population growth rate) and across the three assumptions for future changes in *K*, harvest strategies that met our management objective of maintaining abundance above MNPL ranged from a starting harvest level $H\left(t=1\right)$ = 33 bears/yr at low risk tolerance to $H\left(t=1\right)$ = 85bears/yr at high risk tolerance (Table 1, Fig. 3). A starting harvest of 85 bears/yr was achieved under assumption K2 because, when risk tolerance was high, the relatively rapid decline in K provided more opportunity for compensatory human‐caused removals in early years of projections. However, because we used a state‐dependent framework and simulations had similar starting conditions, faster declines in *K* also led to faster declines in the harvest level. For example, over the 35‐yr projections, the high risk tolerance harvest declined by 46% under assumption *K*1 compared to 66% under *K*2. The highest probabilities of extirpation and male depletion were $P_{\text{ext}}$ ≈ 0.01 and $P_{\text{dep}}$ ≈ 0.07, respectively, for the high risk tolerance harvest strategy under assumption K2 (Table 1).

**Table 1:** Simulation results (set A) for the effects of declining carrying capacity on harvest.

Parameter	Risk tolerance					
	Scenario 1			Scenario 2		
	Low	Medium	High	Low	Medium	High
*K*1						
*F*_0_	0.83	1.27	1.68	0.87	1.24	1.70
*h* ^total^ (*t* = 1)	1.1%	1.7%	2.3%	2.7%	3.9%	5.3%
*H* (*t* = 1)	36	55	72	86	123	169
*H* (*t* = 18)	32	47	59	79	96	91
*H* (*t* = 36)	28	37	39	70	85	78
${\bar{H}}_{\text{yield}}$	36	52	63	84	106	116
*P*_ext_	0.00	0.00	0.00	0.00	0.01	0.07
*P*_dep_	0.00	0.01	0.03	0.01	0.03	0.07
*K*2						
*F*_0_	0.77	1.39	1.96	0.76	1.24	1.75
*h* ^total^ (*t* = 1)	1.0%	1.9%	2.7%	2.3%	3.9%	5.4%
*H* (*t* = 1)	33	60	85	74	123	174
*H* (*t* = 18)	27	48	61	65	92	87
*H* (*t* = 36)	19	29	29	48	66	59
${\bar{H}}_{\text{yield}}$	31	52	64	68	100	111
*P*_ext_	0.00	0.00	0.01	0.00	0.01	0.08
*P*_dep_	0.01	0.03	0.07	0.01	0.05	0.10
*K*3						
*F*_0_	0.76	1.24	1.69	0.87	1.25	1.74
*h* ^total^ (*t* = 1)	1.0%	1.7%	2.3%	2.7%	3.9%	5.4%
*H* (*t* = 1)	33	53	73	86	124	172
*H* (*t* = 18)	30	47	58	81	98	90
*H* (*t* = 36)	27	39	42	76	92	83
${\bar{H}}_{\text{yield}}$	34	53	65	89	111	120
*P*_ext_	0.00	0.00	0.00	0.00	0.01	0.08
*P*_dep_	0.00	0.00	0.03	0.00	0.02	0.07

Notes

The three assumptions for future carrying capacity (*K*1, *K*2, and *K*3) are defined in *Simulations*. The reported harvest strategies met our management objective at low, medium, and high risk tolerances, defined as allowing a 10%, 30%, or 50% probability, respectively, of subpopulation abundance falling below maximum net productivity level. Results reflect a 10‐yr management interval, the baseline level of precision in population data (i.e., *rsd.mod* = 1.0), and a 2:1 male‐to‐female harvest ratio, under vital rate scenarios 1 and 2. The total harvest rate *h* ^total^ (*t*) is the percentage of median total abundance (i.e., independent bears and dependent young of both sexes) removed annually. Other parameters are defined in *Materials and Methods*.

Under vital rate scenario 2 (higher population growth rate) and across the three assumptions for future changes in K, strategies meeting our management objective ranged from $H\left(t=1\right)$ = 74 bears/yr at low risk tolerance to $H\left(t=1\right)$ = 174 bears/yr at high risk tolerance (Table 1). Patterns in results were like scenario 1, except that some harvest strategies were associated with substantive demographic risk as reflected by values of $P_{\text{ext}}$ and $P_{\text{dep}}$ up to 0.10. For example, under assumption K2, a high‐risk tolerance strategy with $H\left(t=1\right)$ = 174 bears/yr was associated with $P_{\text{ext}}$ = 0.08 and $P_{\text{dep}}$ = 0.10. These outcomes were the result of interactions between higher harvest rates and sampling uncertainty.

#### Set B: frequency and precision of new population data

More frequent and precise population data (i.e., smaller values of *mg.int* and *rsd.mod*) led to increased sustainable harvest and long‐term yield. We present results from set B of simulations as proportional changes in sustainable harvest under different combinations of *mg.int* and *rsd.mod*, relative to a baseline harvest strategy with *mg.int* = 10 yr and *rsd.mod* = 1.0 (Table 2). For example, under scenario 1 at medium risk tolerance, the sustainable harvest level at *t* = 18 was 32% higher for simulations with *mg.int* = 5 yr and *rsd.mod* = 0.50, compared to the baseline. Scenarios 1 and 2 exhibited similar patterns in results. Similar improvements to long‐term yield (${\bar{H}}_{\text{yield}}$) were achieved by reducing *mg.int* from 10 to 5 yr or by increasing the precision of population data by changing *rsd.mod* from 1.0 to 0.25. Combinations of more frequent and precise population data outperformed changes to only one factor.

**Table 2:** Simulation results (set B) for how the frequency and precision of population data affect harvest.

*rsd.mod*	*mg.int* (yr)					
	Scenario 1			Scenario 2		
	5	10	15	5	10	15
*H* (*t* = 1)						
0.25	−0.24	−0.27	−0.29	−0.24	−0.28	−0.33
0.50	−0.16	−0.20	−0.24	−0.15	−0.21	−0.26
1.00	0.11	0.00	−0.07	0.19	0.00	−0.12
*H* (*t* = 18)						
0.25	0.43	0.36	0.32	0.38	0.33	0.22
0.50	0.32	0.28	0.19	0.31	0.26	0.17
1.00	0.06	0.00	−0.11	0.08	0.00	−0.06
*H* (*t* = 36)						
0.25	0.67	0.56	0.44	0.38	0.32	0.20
0.50	0.53	0.39	0.31	0.33	0.26	0.13
1.00	0.22	0.00	−0.08	0.15	0.00	−0.11
${\bar{H}}_{\text{yield}}$						
0.25	0.23	0.12	0.02	0.20	0.10	−0.03
0.50	0.19	0.10	−0.02	0.17	0.09	−0.02
1.00	0.12	0.00	−0.08	0.08	0.00	−0.07

Notes

Cells show the proportional change in demographic outcomes as a function of the management interval (*mg.int* = 5, 10, and 15 yr) and precision in population data (*rsd.mod* = 0.25, 0.50, and 1.00), relative to a baseline harvest strategy with an *mg.int* = 10 yr and *rsd.mod* = 1.00. Positive values indicate improvement from the baseline. Results reflect medium risk‐tolerance harvest strategies, defined as allowing a 30% probability of subpopulation abundance falling below maximum net productivity level. Projections used assumption *K*1 for future carrying capacity and a 2:1 male‐to‐female harvest ratio for vital rate scenarios 1 and 2. Parameters are defined in *Materials and Methods*. Corresponding probabilities of male depletion and extirpation are provided in Appendix S2: Table S1.

#### Set C: harvest sex ratio

Increasing the male‐to‐female sex ratio in the harvest (SR) led to moderate increases in sustainable harvest. For example, under scenario 1 at medium risk tolerance, $H\left(t=1\right)$ was ˜11% higher and $H\left(t=36\right)$ was ˜27% higher for SR = 2 compared to SR = 1 (Table 3). Scenarios 1 and 2 exhibited similar patterns in results, with larger values of SR leading to higher sustainable harvest levels, but also to higher probabilities of male depletion (*P*_dep_).

**Table 3:** Simulation results (set C) for the harvest sex ratio.

Parameter	Risk tolerance					
	Scenario 1			Scenario 2		
	Low	Medium	High	Low	Medium	High
SR = 1.0						
*F*_0_	1.11	1.71	2.27	1.08	1.56	1.94
*h* ^total^ (*t* = 1)	1.0%	1.5%	2.1%	2.3%	3.2%	4.0%
*H* (*t* = 1)	31	49	66	72	103	128
*H* (*t* = 18)	24	36	42	58	77	82
*H* (*t* = 36)	21	27	29	49	64	66
${\bar{H}}_{\text{yield}}$	29	43	51	65	86	98
*P*_ext_	0.00	0.00	0.00	0.00	0.01	0.02
*P*_dep_	0.00	0.00	0.00	0.00	0.00	0.01
SR = 1.5						
*F*_0_	0.96	1.46	1.95	0.99	1.38	1.86
*h* ^total^ (*t* = 1)	1.1%	1.6%	2.2%	2.6%	3.5%	4.8%
*H* (*t* = 1)	35	52	70	82	113	154
*H* (*t* = 18)	29	42	52	73	91	91
*H* (*t* = 36)	24	33	37	64	79	81
${\bar{H}}_{\text{yield}}$	33	48	58	78	100	113
*P*_ext_	0.00	0.00	0.00	0.00	0.01	0.04
*P*_dep_	0.00	0.01	0.02	0.00	0.01	0.04

Notes

Demographic outcomes for two alternative harvest sex ratios (SR = 1.0 and 1.5). The reported harvest strategies met our management objective at low, medium, and high risk tolerances, defined as allowing a 10%, 30%, or 50% probability, respectively, of subpopulation abundance falling below maximum net productivity level. Results reflect assumption *K*1 for future carrying capacity, a 10‐yr management interval, and the baseline level of precision in population data (i.e., *rsd.mod* = 1.0), under vital rate scenarios 1 and 2. Results for these inputs with SR = 2.0 are presented in Table 1 and not repeated here. Parameters are defined in *Materials and Methods*.

#### 1 Supplemental simulations

Simulations identical to those in the main text, but with an upper limit of ˜8% on *h* ^total^, led to moderately higher demographic risk when vital rate estimates were imprecise (i.e., when *rsd.mod* = 1). Otherwise, patterns in results were similar (see *Discussion* and Appendix S2).

## Discussion

Our work was motivated by a species that exemplifies how climate change can magnify the challenges of balancing multiple management objectives, which for polar bears often include protecting population viability and providing opportunities for use. Although we focused on a demographic management objective, other types of objectives are possible, such as reducing human–polar‐bear conflicts or protecting human safety. In situations where subsistence need has been quantified, the probability that harvest will meet needs could be included as a quantitative output (IWC 2018). For polar bears and similar species, risk assessment methods that incorporate climate change, use population‐specific data, and articulate clear management objectives will become increasingly important as baselines shift and one‐size‐fits‐all management solutions cease to apply.

An advantage of our framework compared to earlier harvest risk‐assessment methods for polar bears (Taylor et al. 2006) is the ability to consider the demographic effects of habitat loss. Declining sea ice is the primary threat to polar bears throughout their range (Atwood et al. 2016, Regehr et al. 2016). In the CS region, the open‐water period increased by 80 d between 1979 and 2014 (Serreze et al. 2016). We modeled habitat change using a biologically meaningful proxy for *K* derived from remotely sensed sea‐ice data (Stern and Laidre 2016). We used a single, unstructured proxy for *K* even though some CS bears spend the summer and autumn on land while others remain on the sea ice as it retreats northward into the polar basin (Rode et al. 2015), because both strategies can lead to declines in body condition (Whiteman et al. 2015) and their relative effects on CS bears are not well understood. Assumptions for slower (*K*1) and faster (*K*2) declines in future carrying capacity corresponded to reductions in *K* of 3% and 9% per decade, respectively. Our findings demonstrate that, under state‐dependent management, a primary effect of declining *K* is that harvest levels must decline to avoid heightened demographic risk. Although harvest levels declined in our simulations, habitat loss did not require commensurate reductions in the harvest rate because compensatory effects allowed removal of some animals that, in the absence of harvest, would have died from crowding and competition.

Sea‐ice loss has spatial and temporal components (Stern and Laidre 2016) and therefore could affect polar bears through density‐dependent and density‐independent mechanisms, which have different demographic ramifications (Regehr et al. 2021*a*). If declines in *K* are accompanied by habitat‐mediated declines in *r*_MNPL_ and *r*_max_, both the harvest level and the harvest rate will need to be reduced to meet criteria for sustainability. Possible density‐independent effects include mortality associated with increased open water and long‐distance swims (Durner et al. 2011, Pagano et al. 2012). Similarly, for polar bear subpopulations that spend the summer on land, the impacts of longer seasonal fasts could be independent of population density (Pilfold et al. 2016), although this may be less applicable for the CS subpopulation because some bears on land have access to food, such as the beach‐cast carcasses of large whales (Laidre et al. 2018). Using dynamic energy budget models that forecast polar bear recruitment and survival based on the projected duration of seasonal fasts, Molnár et al. (2020) estimated that CS bears will experience demographic declines around the year 2040. We suggest that integrating models of demography and energetics (Robbins et al. 2012, Molnár et al. 2020) is an important area of future research.

Our findings can be used in cost‐benefit analyses for research and management planning. Shorter management intervals and more precise population data reduce risk for a given harvest rate (or increase harvest rate for a given risk level). For example, under vital rate scenario 2, reducing the relative standard deviation of estimated parameters by 50% (i.e., specifying *rsd.mod* = 0.50) led to a 9% increase in long‐term yield (Table 2). We note that lower starting harvest levels associated with smaller values of *rsd.mod* were compensated for by higher sustainable harvests during subsequent management intervals. We did not consider decomposing the management interval into component parts (e.g., to reflect delays between the availability of new population data and changes to the harvest) because such details are difficult to predict and management decisions for the CS subpopulation are made annually (see *Conclusions*).

Male‐biased harvest is a common management tool for polygynous species (Taylor et al. 2008). We found that increasing the male‐to‐female ratio in removals led to moderate increases in sustainable harvest. The demographic advantages of removing male bears, which have lower reproductive value than females, were offset by Allee effects that reduced reproduction as males declined. This finding should be interpreted with caution because we used a generalized model for Allee effects (Molnár et al. 2014), which appear to explain observed demographic trends in some other polar bear subpopulations (Molnár et al. 2008, 2014) but have not been documented for CS bears.

We used the first quantitative estimates of vital rates and abundance for the CS polar bear subpopulation to parameterize the matrix model at the start of projections. The estimate of abundance from the CS‐IPM (mode = 2,937; Regehr et al. 2018) was lower than, but of similar magnitude to, independent estimates of abundance from a 2016 aerial survey (range of point estimates = 3,435–5,444, depending on the proportion of bears assumed to be missed on the transect line; Conn et al. 2021). For vital rate scenario 1, the estimate *r*_MNPL_ = 0.02 suggests a limited capacity for growth compared to other polar bear subpopulations (Regehr et al. 2017). Interpretation of results is complicated by potential negative bias in survival and lack of direct information on the relative density of the CS subpopulation (e.g., if relative density was higher than assumed, low survival estimates could reflect density‐dependent regulation rather than limited growth potential). Nonetheless, scenario 1 is relevant because it represents a polar bear subpopulation with reduced capacity for growth, which is a potential consequence of habitat loss if it affects population dynamics through density‐independent mechanisms.

Under vital rate scenario 2, the estimate *r*_MNPL_ = 0.04 was, by design, typical for polar bears (Regehr et al. 2017). Several lines of evidence suggest that scenario 2 is a more plausible representation of the CS subpopulation from 2008–2016. The status of ice‐dependent seals in the region has been positive (Crawford et al. 2015) and CS bears have maintained access to prey during the critical spring foraging period (Rode et al. 2018). Body condition and reproductive indices have not declined since the 1980s, despite sea‐ice loss (Rode et al. 2014, 2021, Regehr et al. 2018). Finally, a pilot IK study designed to inform quantitative modeling (Braund et al. 2018) provided results consistent with previous IK (Voorhees et al. 2014) and the assumptions inherent to scenario 2. Although growth rates well above *r*_MNPL_ = 0.04 are possible for polar bears in biologically productive regions (Regehr et al. 2017), we did not consider survival rates higher than those in scenario 2.

The management objective of maintaining the equilibrium size of a harvested subpopulation above MNPL is intended to keep abundance on the right (i.e., conservative) side of the harvest yield curve (USFWS 2016). Because MNPL is defined relative to *K*, this objective accommodates potential changes in the environment. In some situations, this may be more useful than a management objective that seeks to maintain a constant abundance, which may not be possible under climate change. A general caveat is that any non‐negligible harvest will result in a lower equilibrium abundance. For example, a harvested polar bear subpopulation could be ˜30% below *K* and still meet the management objective. Lower starting abundance could shorten time to extirpation under progressive habitat loss, and possibly preclude recovery if habitat were to stabilize during the “buffer years” that would have been available if starting from a higher, unharvested abundance.

Our estimates of harvest risk can be approached by comparing demographic outcomes for a moderate harvest strategy, under both vital rate scenarios, and then evaluating pros and cons of lower or higher harvests. Under vital rate scenario 2, a low risk‐tolerance strategy had a starting harvest level $H\left(t=1\right)$ = 86 bears/yr (Table 1). This would likely meet our management objective, not have undesired demographic outcomes, and lead to harvest levels that decline gradually over time due to projected declines in *K*. If the demographic status of the CS subpopulation was actually like vital rate scenario 1, which corresponded to a lower population growth rate, a strategy with $H\left(t=1\right)$ = 86 bears/yr would probably not meet our management objective (i.e., *P*_MO_ < 0.50; Table 1). Risk tolerances might be exceeded, and the harvest level would decline faster over time. However, the chances of severe negative outcomes would remain low (i.e., *P*_ext_ < 0.01 and *P*_dep_ < 0.07) and the state‐dependent framework would provide opportunities to adjust the harvest, if necessary.

Implementing a harvest rate that is proportional to *N*, and periodically updating the harvest rate and level using new population data, is more robust than a fixed‐rate or, especially, a fixed‐level harvest strategy (Quinn and Deriso 1999). For the CS subpopulation, we modified the state‐dependent framework proposed by Regehr et al. (2017) by placing an upper limit of ˜5% on *h* ^total^. This reflected the assumption that future harvest rates will generally not exceed 4.5%, the historical standard for polar bears at a 2:1 male‐to‐female ratio when environmental conditions were stable (Taylor et al. 1987). Increasing the upper limit on *h* ^total^ to 8%, which is near the theoretical maximum for polar bears (Regehr et al. 2017), increased demographic risk when using imprecise estimates of *r*_MNPL_ in Equations 1 and 2 (Appendix S2). For example, for set A of simulations under assumption *K*1 for carrying capacity, increasing the upper limit on *h* ^total^ led to an average reduction of 18% in $H\left(t=1\right)$ and 7% in long‐term yield (${\bar{H}}_{\text{yield}}$), when considering harvest strategies that met the management objective at medium risk tolerance (Table 1 and Appendix S2: Table S2). In contrast, for set B of simulations with identical conditions but more precise estimates of *R*_MNPL_ (i.e., using *rsd.mod* = 0.25), increasing the upper limit on *h* ^total^ led to average reductions of only 4% in both $H\left(t=1\right)$ and ${\bar{H}}_{\text{yield}}$ (Table 2 and Appendix S2: Table S3). These comparisons highlight how uncertainty in vital rates contributes to demographic risk.

Vital rate scenario 1 represented a polar bear subpopulation with reduced capacity for growth. However, we did not investigate the possibility of progressive reductions in *r*_MNPL_ over time. Negative density‐independent effects that occur rapidly with respect to the management interval (e.g., a reduced *R*_MNPL_ that is not detected for multiple years), or negative density‐dependent effects that are more severe than our analyses, would lead to higher probabilities of undesired outcomes, including extirpation. A conservative approach to harvest management is one way to reduce demographic risk. Other options include ongoing monitoring of environmental, ecological, or demographic indices that can trigger changes to research or management (e.g., shortening the management interval), or establishing a multi‐level system under which graduated management and conservation actions are tied to pre‐established population thresholds (USFWS 2016).

## Conclusions

In 2018, commissioners of the polar bear agreement between the United States and Russia adopted a harvest level of up to 85 bears/yr, of which no more than one‐third are female (USFWS 2018). This decision was informed by results suggesting that starting harvest levels of 50, 85, and 120 bears/yr corresponded broadly to low, moderate, and high degrees of risk of reducing the CS subpopulation below MNPL, respectively (Tables 1, 2, 3). These harvest levels are equivalent to starting total harvest rates of 1.7% (95% CRI = 0.8–3.2%), 2.7% (95% CRI = 1.4–5.5%), and 3.9% (95% CRI = 2.2–7.8%), respectively. Within this range, the risk of undesired demographic outcomes increases with higher harvest, and the risk of unnecessarily limiting subsistence opportunities increases with lower harvest. Our results point toward total harvest rates below 4.5%, the historical standard for polar bears (Taylor et al. 1987), despite evidence that the CS subpopulation has been productive in recent years. This is because estimates of vital rates and abundance from the CS‐IPM were imprecise and possibly biased (Regehr et al. 2018), and because we considered habitat loss. The harvest strategies reported here would be riskier without state‐dependent management.

We demonstrated use of a population modeling‐management framework to evaluate interactions between harvest, habitat change, future research considerations, and productivity and viability for a wildlife population. Results are a series of potential harvest strategies and forecasted demographic outcomes (i.e., statements of harvest risk). Our findings should be interpreted with caution given that continued warming could result in complex ecological interactions (e.g., between prey status and habitat availability; Stirling 2002) and nonlinear demographic responses as critical thresholds are passed (Molnár et al. 2020). Our methods are intended to inform current management and provide a basis for developing quantitative biological hypotheses against which future demographic and environmental data can be evaluated (Houlahan et al. 2017). Given that the demographic effects of climate change are variable and poorly understood for many species, the combination of demographic risk assessment and state‐dependent harvest management is a powerful framework for balancing protection and use.

## Supporting information

Appendix S1

Appendix S2

Appendix S3

Appendix S4

Appendix S5

Appendix S6

## Data Availability

Code (Regehr et al. 2021*b*) is available in Zenodo: https://doi.org/10.5281/zenodo.5093799
