# Supplementary material for: Demographic risk assessment for a harvested species threatened by climate change: polar bears in the Chukchi Sea
Source: Ecol Appl. 2021 Oct 26;31(8):e02461. doi: 10.1002/eap.2461 (PMC9286533; doi:10.1002/eap.2461)
Supplement: Supplementary file 1 — Appendix S1 [file EAP-31-0-s002.pdf]

Supporting Information. Regehr, E.V., M.C. Runge, A. Von Duyke, R.R. Wilson, L. Polasek, K.D. Rode, N.J. Hostetter, and S.J. Converse. 2021. Demographic risk assessment for a harvested species threatened by climate change: polar bears in the Chukchi Sea. Ecological Applications.

Appendix S1: Allee effects in the mating system

Our matrix projection model was based on Regehr et al. (2017) and incorporated a new mechanistic submodel for Allee effects in the mating system. Molnár et al. (2014) proposed that, under some conditions, reproductive rates for polar bears may decline due to limitations in the ability to find mates. Such declines can occur if adult males are depleted relative to adult females, which is possible under sex-selective harvest (Taylor et al. 2008), or if polar bear densities are low during the breeding season (Molnár et al. 2008). Because Allee effects in the Chukchi Sea (CS) subpopulation have not been studied, we based the Allee submodel on equation 3 in Molnár et al. (2014) with input parameters for a "generic population". Using an area of 815,000 km^2 (excluding land) within the CS subpopulation boundary and estimates of numbers of solitary males and females from the CS-IPM (Regehr et al. 2018), the Allee submodel produced a fertilization probability of 0.85, which is close to the estimate of breeding probability from the CS-IPM (mode = 0.83 [95% CRI = 0.71–0.90] for the parameter β_4 in Figure 2, which represents the probability that an adult female in stage 4 in year t will transition to stage 5 in year $t + 1$, conditional on survival). This suggested that adjustment for mating season aggregation was not required for the Allee submodel to match empirical data on reproduction. During forward projections of the CS subpopulation, we divided the estimated probability of fertilization from the Allee submodel in each year ($t = 2, 3, \ldots 36$) by the estimate from $t = 1$ and constrained the resulting dimensionless parameter to the interval [0,1]. We then incorporated potential Allee effects by multiplying this dimensionless parameter by the value of

β_4 obtained from the density-dependent curves of the vital rates (Appendix S3). Under this approach, the value of β_4 at $t = 1$ was unmodified from its empirical estimate (i.e., there were no Allee effects under initial conditions) and subpopulations only experienced reproductive declines due to Allee effects if a projection resulted in depletion of adult male bears or a substantive reduction in density. It was important to consider Allee effects because, if they were not included, harvest strategies with high harvest rates and selection for male bears could result in subpopulations with unrealistically high growth rates, because most adult animals would be female and breeding probabilities would remain high even in the near absence of sexually mature males. Our representation of Allee effects did not account for changes in sea-ice area during the mating season that could result from future habitat loss.

LITERATURE CITED

Molnár, P. K., A. E. Derocher, M. A. Lewis, and M. K. Taylor. 2008. Modelling the Mating System of Polar Bears: a Mechanistic Approach to the Allee Effect. Proceedings of the Royal Society B-Biological Sciences 275:217-226.

Molnár, P. K., M. A. Lewis, and A. E. Derocher. 2014. Estimating Allee Dynamics before They Can Be Observed: Polar Bears as a Case Study. PLoS ONE 9(1): e85410.

Regehr, E. V., N. J. Hostetter, R. R. Wilson, K. D. Rode, M. S. Martin, and S. J. Converse. 2018. Integrated Population Modeling Provides the First Empirical Estimates of Vital Rates and Abundance for Polar Bears in the Chukchi Sea. Sci. Rep. 8:16780.

Regehr, E. V., R. R. Wilson, K. D. Rode, M. C. Runge, and H. Stern. 2017. Harvesting wildlife affected by climate change: a modeling and management approach for polar bears. J. Appl. Ecol. 54:1534-1543.

Taylor, M. K., P. D. McLoughlin, and F. Messier. 2008. Sex-selective harvesting of polar bears *Ursus maritimus*. Wildlife Biology 14:52-60.
