# Supplementary material for: Demographic risk assessment for a harvested species threatened by climate change: polar bears in the Chukchi Sea
Source: Ecol Appl. 2021 Oct 26;31(8):e02461. doi: 10.1002/eap.2461 (PMC9286533; doi:10.1002/eap.2461)
Supplement: Supplementary file 2 — Appendix S2 [file EAP-31-0-s005.pdf]

Supporting Information. Regehr, E.V., M.C. Runge, A. Von Duyke, R.R. Wilson, L. Polasek, K.D. Rode, N.J. Hostetter, and S.J. Converse. 2021. Demographic risk assessment for a harvested species threatened by climate change: polar bears in the Chukchi Sea. Ecological Applications.

Appendix S2: Supplemental simulations and results

In the main text, set B of simulations evaluated how the frequency and precision of new population data affect demographic risk. Results are presented as proportional changes in sustainable harvest under different combinations of management interval (*mg.int*) and level of precision (*rsd.mod*), relative to a baseline harvest strategy with *mg.int* = 10 years and *rsd.mod* = 1.0 (Table 2). Table S1 provides additional results for set B of simulations.

All simulations in the main text imposed an upper limit of 0.10 on $h(t)$, the harvest rate at each timestep referenced to the 15th percentile of the sampling distribution for abundance of independent bears. This corresponded to an upper limit of approximately 5% on the total harvest rate, h^{total}, defined as the percentage of total abundance, including independent bears and dependent young of both sexes, removed annually. The constraint on h was intended to mitigate risk associated with large uncertainty in estimates of finite growth rate (i.e., R_{MNPL}) from the integrated population model for Chukchi Sea polar bears (Regehr et al. 2018). To evaluate the effects of this constraint, we repeated the three sets of simulations described in the main text (see *Simulations*) under identical conditions but using a higher upper limit of 0.17 on h, which corresponded to a limit of approximately 8% on h^{total}. Comparison of results in Tables S2, S3, and S4 (using an upper limit of 8% on h^{total}) with results in Tables 1, 2, and 3 (using an upper limit of 5% on h^{total}) demonstrates that constraining the maximum harvest rate can reduce demographic risk resulting from sampling uncertainty in vital rate estimates. For example, across all simulations in set A, increasing the upper limit on h^{total} from 5% to 8% required average

reductions of 22% and 10% in the starting harvest level ($H[t = 1]$) and long-term yield ($\overline{H}_{yield}$), respectively, to avoid increasing demographic risk.

LITERATURE CITED

Regehr, E. V., N. J. Hostetter, R. R. Wilson, K. D. Rode, M. S. Martin, and S. J. Converse. 2018. Integrated Population Modeling Provides the First Empirical Estimates of Vital Rates and Abundance for Polar Bears in the Chukchi Sea. Sci. Rep. 8:16780.

TABLES

Table S1. Additional results for set B of simulations on how the frequency and precision of population data affect harvest. Cells present the probabilities of extirpation (P_{ext}) and male depletion (P_{dep}), corresponding to the same harvest strategies for which demographic outcomes are reported in Table 2. The management interval (*mg.int* = 5, 10, and 15 years) and precision in population data (*rsd.mod* = 0.25, 0.50, and 1.00) varied across simulations. Projections used assumption $K1$ for future carrying capacity and a 2:1 male-to-female harvest ratio, with vital rates from scenarios 1 and 2. Results reflect medium risk-tolerance harvest strategies, defined as allowing a 30% probability of subpopulation abundance falling below maximum net productivity level. Parameters are defined in the main text.

			scenario 1				scenario 2		
			$mg.int$ (years)				$mg.int$ (years)		
			5	10	15		5	10	15
			P_{ext}						
rsd.mod	0.25		0.00	0.00	0.00		0.00	0.00	0.01
	0.50		0.00	0.00	0.00		0.00	0.00	0.01
	1.00		0.00	0.00	0.00		0.00	0.01	0.03
			P_{dep}						
rsd.mod	0.25		0.01	0.02	0.05		0.01	0.02	0.04
	0.50		0.01	0.02	0.03		0.01	0.02	0.04
	1.00		0.00	0.01	0.03		0.01	0.03	0.05

Table S2. Simulation results (set A) for the effects of declining carrying capacity on harvest, using an upper limit of approximately 8% on h^{total}, the proportion or percentage of total abundance (i.e., independent bears and dependent young of both sexes) removed annually. Comparable simulations in the main text (Table 1) used an upper limit of approximately 5% on h^{total}. The three assumptions for future carrying capacity ($K1$, $K2$, and $K3$) are defined in the main text. The reported harvest strategies met our management objective at low, medium, and high-risk tolerances, defined as allowing a 10%, 30%, or 50% probability, respectively, of subpopulation abundance falling below maximum net productivity level. Results reflect a 10-year management interval, the baseline level of precision in population data (i.e., *rsd.mod* = 1.0), and a 2:1 male-to-female harvest ratio, under vital rate scenarios 1 and 2. Parameters are defined in the main text.

		scenario 1				scenario 2		
		risk tolerance						
		low	medium	high		low	medium	high
$K1$	F_O	0.74	1.12	1.43		0.73	1.01	1.21
	$h^{total}(t=1)$	1.0%	1.5%	1.9%		2.3%	3.1%	3.8%
	$H(t=1)$	32	49	62		72	100	120
	$H(t=18)$	28	43	53		67	92	106
	$H(t=36)$	23	31	31		59	71	69
	$\overline{H}_{yield}$	32	48	59		73	100	113
	P_{ext}	0.00	0.01	0.03		0.00	0.01	0.04
	P_{dep}	0.01	0.04	0.08		0.02	0.06	0.11
$K2$	F_O	0.66	1.17	1.59		0.61	1.01	1.24
	$h^{total}(t=1)$	0.9%	1.6%	2.1%		1.9%	3.1%	3.9%
	$H(t=1)$	28	51	68		61	100	123
	$H(t=18)$	22	43	57		52	88	105
	$H(t=36)$	15	23	22		38	54	51
	$\overline{H}_{yield}$	26	47	60		56	91	107
	P_{ext}	0.01	0.02	0.04		0.01	0.02	0.06

	P_{dep}	0.02	0.07	0.11		0.02	0.08	0.15
$K3$	F_O	0.71	1.09	1.40		0.75	1.02	1.22
	$h^{total}(t=1)$	0.9%	1.5%	1.9%		2.3%	3.2%	3.8%
	$H(t=1)$	30	48	60		74	101	121
	$H(t=18)$	27	42	53		72	95	109
	$H(t=36)$	24	33	34		66	80	78
	$\overline{H}_{yield}$	31	48	60		79	104	118
	P_{ext}	0.00	0.01	0.02		0.00	0.01	0.04
	P_{dep}	0.01	0.03	0.07		0.01	0.05	0.11

Table S3. Simulation results (set B) for how the frequency and precision of population data affect harvest, using an upper limit of approximately 8% on h^{total}, the proportion or percentage of total abundance (i.e., independent bears and dependent young of both sexes) removed annually. Comparable simulations in the main text (Table 2) used an upper limit of approximately 5% on h^{total}. Cells show the proportional change in demographic outcomes as a function of the management interval (*mg.int* = 5, 10, and 15 years) and precision in population data (*rsd.mod* = 0.25, 0.50, and 1.00), relative to a baseline harvest strategy with a *mg.int* = 10 years and *rsd.mod* = 1.00. Positive values indicate improvement from the baseline. Results reflect medium risk-tolerance harvest strategies, defined as allowing a 30% probability of subpopulation abundance falling below maximum net productivity level. Projections used assumption $K1$ for future carrying capacity and a 2:1 male-to-female harvest ratio for vital rate scenarios 1 and 2. Parameters are defined in the main text.

		scenario 1			scenario 2		
		mg.int (years)			*mg.int* (years)		
		5	10	15	5	10	15
		$H(t=1)$					
rsd.mod	0.25	-0.17	-0.19	-0.21	-0.10	-0.16	-0.19
	0.50	-0.08	-0.12	-0.15	-0.05	-0.11	-0.15
	1.00	0.06	0.00	-0.04	0.10	0.00	-0.05
		$H(t=18)$					
rsd.mod	0.25	0.51	0.42	0.40	0.43	0.33	0.26
	0.50	0.37	0.33	0.28	0.32	0.22	0.16
	1.00	0.02	0.00	-0.05	0.06	0.00	-0.06
		$H(t=36)$					
rsd.mod	0.25	0.84	0.68	0.61	0.61	0.47	0.39
	0.50	0.65	0.52	0.45	0.50	0.36	0.24
	1.00	0.19	0.00	-0.06	0.20	0.00	-0.04
		$\overline{H}_{yield}$					
rsd.mod	0.25	0.27	0.17	0.06	0.25	0.13	0.03
	0.50	0.27	0.17	0.04	0.21	0.09	-0.01
	1.00	0.12	0.00	-0.06	0.10	0.00	-0.08

Table S4. Simulation results (set C) for the harvest sex ratio, using an upper limit of approximately 8% on h^{total}, the proportion or percentage of total abundance (i.e., independent bears and dependent young of both sexes) removed annually. Comparable simulations in the main text (Table 3) used an upper limit of approximately 5% on h^{total}. Demographic outcomes for two alternative harvest sex ratios (SR = 1.0 and 1.5). The reported harvest strategies met our management objective at low, medium, and high-risk tolerances, defined as allowing a 10%, 30%, or 50% probability, respectively, of subpopulation abundance falling below maximum net productivity level. Results reflect assumption $K1$ for future carrying capacity, a 10-year management interval, and the baseline level of precision in population data (i.e., *rsd.mod* = 1.0), under vital rate scenarios 1 and 2. Results for these inputs with SR = 2.0 are presented in Table S2 and not repeated here. Parameters are defined in the main text.

		scenario 1				scenario 2		
		low	medium	high		low	medium	high
SR = 1.0	F_O	1.04	1.59	2.06		0.99	1.41	1.70
	$h^{total}(t=1)$	29	46	60		66	93	112
	$H(t=1)$	0.9%	1.4%	1.9%		2.1%	2.9%	3.5%
	$H(t=18)$	23	34	41		55	72	82
	$H(t=36)$	18	26	26		46	58	60
	$\overline{H}_{yield}$	27	41	49		61	82	96
	P_{ext}	0.00	0.01	0.02		0.00	0.01	0.03
	P_{dep}	0.00	0.01	0.02		0.00	0.01	0.02
SR = 1.5	F_O	0.83	1.32	1.70		0.85	1.18	1.40
	$h^{total}(t=1)$	30	47	61		71	98	115
	$H(t=1)$	0.9%	1.5%	1.9%		2.2%	3.1%	3.6%
	$H(t=18)$	25	38	48		64	87	97
	$H(t=36)$	21	29	29		56	70	72
	$\overline{H}_{yield}$	29	45	55		70	94	107
	P_{ext}	0.00	0.01	0.02		0.00	0.01	0.03
	P_{dep}	0.01	0.03	0.05		0.01	0.03	0.06
