# Supplementary material for: Demographic risk assessment for a harvested species threatened by climate change: polar bears in the Chukchi Sea
Source: Ecol Appl. 2021 Oct 26;31(8):e02461. doi: 10.1002/eap.2461 (PMC9286533; doi:10.1002/eap.2461)
Supplement: Supplementary file 3 — Appendix S3 [file EAP-31-0-s006.pdf]

**Supporting Information.** Regehr, E.V., M.C. Runge, A. Von Duyke, R.R. Wilson, L. Polasek, K.D. Rode, N.J. Hostetter, and S.J. Converse. 2021. Demographic risk assessment for a harvested species threatened by climate change: polar bears in the Chukchi Sea. Ecological Applications.

**Appendix S3: Operations during population projections**

We evaluated harvest risk by projecting polar bear subpopulations 35 years into the future (i.e., from $t = 1, 2, \ldots 36$), which is equivalent to approximately three polar bear generations (Regehr et al. 2016). This timeframe reduced the impact of transient dynamics at the start of projections and allowed assessment of the long-term effects of harvest. At each time step the following operations were performed.

1. Simulated subpopulations were projected forward one year using the stage-structured matrix model: $\boldsymbol{n}(t+1) = \boldsymbol{A}(t) \times \boldsymbol{n}(t)$, where $\boldsymbol{n}(t)$ is a stage distribution vector representing the number of animals in each life-cycle stage at time step $t$, and $\boldsymbol{A}(t)$ is a 10×10 projection matrix. Entries in $\boldsymbol{A}(t)$ were defined in terms of vital rates in the life cycle graph (Figure 2).
2. The harvest level calculated from equations 1 and 2 was allocated among life cycle stages using a multinomial distribution with the probability for each stage calculated as the product of its proportional stage distribution and harvest vulnerability vector (see below).
3. Relative density of the simulated subpopulation was determined as the sum of metabolic energetic equivalent (*mee*) values in the subpopulation, divided by $K$ expressed as energetic equivalents, using the methods of Regehr et al. (2017). Calculating relative density based on energetic requirements, rather than simple numbers of bears, allows animals of different sizes, and thus different nutritional requirements, to have a different contribution to density effects.

4. Vital rates corresponding to the current relative density were determined from the density-dependent curves, with modifications applied to the parameter $\beta_4$ based on the Allee submodel. These vital rates were used to construct a projection matrix for the next time step $\boldsymbol{A}(t+1)$.

For a given projection, the harvest strategy was defined by specifying values for the management parameters $F_O$, $SR$, *mg.int*, and *rsd.mod* (see *State-dependent harvest management*). The annual harvest level during the first management interval (i.e., years *t* = 2,3,…, *mg.int* + 1) was calculated with equations 1 and 2 using biological parameters (i.e., values of $\tilde{R}_{MNPL}$ and $\tilde{N}$) estimated from the CS-IPM for the period 2008–2016 (Regehr et al. 2018) as represented by vital rate scenario 1 or 2. This ensured that harvest levels during the first management interval were calculated based on currently available information and were consistent across projections with the same harvest strategy.

At the beginning of subsequent management intervals, the harvest level was calculated using time-constant values of the management parameters (*mg.int* and *rsd.mod*) with values of $\tilde{R}_{MNPL}$ and $\tilde{N}$ derived from simulated research and monitoring studies. The notation $(t)$ in equations 1 and 2 identifies parameters that are periodically updated under the state-dependent approach. Conceptually, the simulated research studies represent new field investigations, performed in the future, to obtain updated estimates of vital rates and abundance. The simulated research studies selected values of $\tilde{R}_{MNPL}$ and $\tilde{N}$ from a multivariate normal distribution with a covariance structure calculated from the CS-IPM, as described in the main text.

Throughout our analyses, harvest level refers to the number of independent bears removed from the CS subpopulation by humans each year (i.e., the combination of subsistence

harvest, defense kills, etc.). To account for selectivity in human-caused removals (Schliebe et al. 2016) and individual variation in the reproductive value of polar bears (Hunter et al. 2007), harvest was implemented using stage-specific harvest vulnerability vectors. For females and males separately, we estimated harvest vulnerability by comparing the stage structure of the observed harvest from the CS subpopulation in Alaska and Chukotka (Appendix S5) to the mean stage structure of the subpopulation as estimated from the CS-IPM. The resulting harvest vulnerability vectors for females (stages 1–6) and males (stages 7–10) were [0.72, 0.72, 1.0, 1.0, 0.14, 0.14] and [2.17, 2.17, 2.17, 1.0], respectively (e.g., a subadult male in stage 7 was 2.17 times as likely to be harvested than an adult male in stage 10). During some projections, selective harvest led to depletion of bears in one or more stages. If the specified harvest level exceeded the number of bears in a stage, excess harvest was applied to adult bears of the same sex (i.e., stages 4 or 10). If the harvest exceeded the total number of males or females, excess harvest was applied to adults of the other sex. The harvest vulnerability vectors remained constant across projections and timesteps. During population projections, harvest was applied deterministically such that the exact harvest level rounded to the nearest bear, as calculated from equations 1 and 2, was removed from the subpopulation each year.

LITERATURE CITED

Hunter, C. M., H. Caswell, M. C. Runge, S. C. Amstrup, E. V. Regehr, and I. Stirling. 2007. Polar bears in the southern Beaufort Sea II: demography and population growth in relation to sea ice conditions. USGS Alaska Science Center, Anchorage, Administrative Report, iv + 46 pp.

Regehr, E. V., N. J. Hostetter, R. R. Wilson, K. D. Rode, M. S. Martin, and S. J. Converse. 2018. Integrated Population Modeling Provides the First Empirical Estimates of Vital Rates and Abundance for Polar Bears in the Chukchi Sea. Sci. Rep. 8:16780.

Regehr, E. V., K. Laidre, H. R. Akçakaya, S. Amstrup, T. Atwood, N. Lunn, M. Obbard, H. Stern, G. Thiemann, and Ø. Wiig. 2016. Conservation status of polar bears (*Ursus maritimus*) in relation to projected sea-ice declines. Biol. Lett. 12.

Regehr, E. V., R. R. Wilson, K. D. Rode, M. C. Runge, and H. Stern. 2017. Harvesting wildlife affected by climate change: a modeling and management approach for polar bears. J. Appl. Ecol. 54:1534-1543.

Schliebe, S., B. Benter, E. V. Regehr, L. Quakenbush, J. Omelak, M. Nelson, and K. Nesvacil. 2016. Co-management of the Alaskan harvest of the Alaska–Chukotka polar bear subpopulation: How to implement a harvest quota. Wildlife Technical Bulletin ADF&G/DWC/WTB-2016-15, Division of Wildlife Conservation, Alaska Department of Fish and Game, Juneau, Alaska.
