# Supplementary material for: Demographic risk assessment for a harvested species threatened by climate change: polar bears in the Chukchi Sea
Source: Ecol Appl. 2021 Oct 26;31(8):e02461. doi: 10.1002/eap.2461 (PMC9286533; doi:10.1002/eap.2461)
Supplement: Supplementary file 4 — Appendix S4 [file EAP-31-0-s001.pdf]

Supporting Information. Regehr, E.V., M.C. Runge, A. Von Duyke, R.R. Wilson, L. Polasek, K.D. Rode, N.J. Hostetter, and S.J. Converse. 2021. Demographic risk assessment for a harvested species threatened by climate change: polar bears in the Chukchi Sea. Ecological Applications.

Appendix S4: Assumptions for changing carrying capacity

For use in population projections, we derived a proxy metric to represent potential changes in environmental carrying capacity (K) using satellite data for sea-ice area. Specifically, we used the number of "ice-covered days" within the CS subpopulation boundary, calculated using the methods of Stern and Laidre (2016) from the Sea Ice Concentrations from Nimbus-7 SMMR and DMSP SSM/I-SSMIS Passive Microwave Data (Cavalieri et al., 1996) data set available from the National Snow and Ice Data Center (NSIDC) in Boulder, Colorado, USA. Each year the sea-ice area reaches a maximum in March and a minimum in September. Sea-ice area is defined as sea-ice concentration × grid cell area, summed over cells with sea-ice concentration greater than 15%. A threshold was defined halfway between the mean March sea-ice area and the mean September sea-ice area for the period 1979–2016. Then, the number of ice-covered days was calculated as the total number of days between when the sea-ice area drops below the threshold in spring and rises above the threshold in fall. To represent future trends and variability in K, we fit linear models to the observed time series of ice-covered days, and then projected the metric forward in time using the methods of Gelman and Hill (2007) to simulate uncertainty in the slope and residual standard errors. Finally, we standardized the metric by dividing the projected values of ice-covered days at year $t = 2, 3, \ldots 36$ by the fitted value at year $t = 1$. This resulted in a dimensionless parameter (κ) representing proportional changes in K. During projections, carrying capacity at year t, calculated as $K(t) = K(t = 1) \times \kappa(t)$, operated on the vital rates through density-dependent curves generated using the methods of Regehr et al. (2017). This modeling approach reflected the assumption that polar bear vital rates are affected

by habitat change exclusively through density-dependent mechanisms. We represented future habitat by projecting an empirical sea-ice metric based on remote-sensing data, rather than using forecasts from general circulation models (GCMs; SIMIP Community [2020]), because the models have coarse spatiotemporal resolution compared to the relatively limited geographic area and duration of our projections.

It is not possible to accurately forecast the status of the CS subpopulation under climate change based on existing information. Therefore, we performed population projections to evaluate harvest risk under three plausible assumptions for future carrying capacity. Under the third assumption (denoted $K3$ in the main text), K remained stable until 2036 and then declined. We subjectively selected 2036 as the transition year based on forecasted sea-ice conditions in the CS region using the methods, GCMs (n = 6), and representative concentration pathways (RCPs, n = 2) described in Douglas and Atwood (2017). Specifically, by the year 2036, for both RCP = 4.5 and 8.5, at least one GCM indicated that sea ice would be farther than 200 km away from the coastlines of Wrangel Island and Chukotka, the most important summering area for CS polar bears (Rode et al. 2015), for more than four months per year, in at least six of the 10 years during the period 2036–2045. Dynamic energy budget models have been used to estimate the approximate period of food deprivation beyond which polar bears are expected to experience declines in reproduction and survival (Molnár et al. 2010, 2011, 2020; Robbins et al. 2012). Estimates for CS bears suggest that reduced adult survival would be possible, and reduced cub recruitment likely, around the year 2040 (Figure 4 in Molnár et al. 2020). This forecasted timeframe for the onset of negative demographic effects resulting from habitat loss is similar to our assumption $K3$ for future carrying capacity. Furthermore, we viewed $K3$ as broadly consistent with perspectives from some Indigenous Knowledge holders that climate change

affects the year-to-year demographic status of polar bears but has not yet resulted in subpopulation declines, although it remains possible that declines will occur in the future (Vorhees et al. 2014; Braund et al. 2018).

LITERATURE CITED

Braund, S. R., P. B. Lawrence, E. G. Sears, R. K. Schraer, B. Adams, T. Hepa, J.C. George, and A.L. Von Duyke. 2018. Polar Bear TEK: A Pilot Study to Inform Polar Bear Management Models. North Slope Borough Department of Wildlife Management Research Report: NSB.DWM.RR.2018-01. Utqiaġvik, Alaska USA.

Cavalieri, D. J., Parkinson, C. L., Gloersen, P., and H.J. Zwally. 1996, updated yearly. Sea Ice Concentrations from Nimbus-7 SMMR and DMSP SSM/I- SSMIS Passive Microwave Data, Version 1, Boulder, Colorado USA, NASA National Snow and Ice Data Center Distributed Active Archive Center, doi:10.5067/8GQ8LZQVL0VL.

Douglas, D. C., and T. C. Atwood. 2017. Uncertainties in forecasting the response of polar bears to global climate change. Pages 463-473 in A. Butterworth, editor. Marine Mammal Welfare. Springer.

Gelman, A., and J. Hill. 2007. Data analysis using regression and multilevel/hierarchical models. Cambridge University Press, New York, New York, USA.

Molnár, P. K., C. M. Bitz, M. M. Holland, J. E. Kay, S. R. Penk, and S. C. Amstrup. 2020. Fasting season length sets temporal limits for global polar bear persistence. Nature Climate Change 10:732.

Molnár, P.K., A.E. Derocher, T. Klanjscek, and M.A. Lewis. 2011. Predicting climate change impacts on polarbear litter size. Nature communications 2:186.

Molnár, P. K., A. E. Derocher, G. W. Thiemann, and M. A. Lewis. 2010. Predicting survival, reproduction and abundance of polar bears under climate change. Biol. Conserv.143:1612-1622.

Regehr, E. V., R. R. Wilson, K. D. Rode, M. C. Runge, and H. Stern. 2017. Harvesting wildlife affected by climate change: a modeling and management approach for polar bears. J. Appl. Ecol. 54:1534-1543.

Robbins, C. T., C. Lopez-Alfaro, K. D. Rode, O. Toien, and O. L. Nelson. 2012. Hibernation and seasonal fasting in bears: the energetic costs and consequences for polar bears. J. Mammal. 93:1493-1503.

Rode, K. D., R. R. Wilson, E. V. Regehr, M. S. Martin, D. C. Douglas, and J. Olson. 2015. Increased land use by Chukchi Sea polar bears in relation to changing sea ice conditions. PLoS ONE:10.1371/journal.pone.0142213.

SIMIP Community. 2020. Arctic sea ice in CMIP6. Geophysical Research Letters, 47, e2019GL086749. https://doi.org/10.1029/2019GL086749.

Stern, H. L., and K. L. Laidre. 2016. Sea-ice indicators of polar bear habitat. The Cryosphere 10:2027-2041.

Voorhees, H., R. Sparks, H. P. Huntington, and K. D. Rode. 2014. Traditional Knowledge about Polar Bears (*Ursus maritimus*) in Northwestern Alaska. Arctic 67:523-536.
