# Supplementary material for: Demographic risk assessment for a harvested species threatened by climate change: polar bears in the Chukchi Sea
Source: Ecol Appl. 2021 Oct 26;31(8):e02461. doi: 10.1002/eap.2461 (PMC9286533; doi:10.1002/eap.2461)
Supplement: Supplementary file 5 — Appendix S5 [file EAP-31-0-s003.pdf]

Supporting Information. Regehr, E.V., M.C. Runge, A. Von Duyke, R.R. Wilson, L. Polasek, K.D. Rode, N.J. Hostetter, and S.J. Converse. 2021. Demographic risk assessment for a harvested species threatened by climate change: polar bears in the Chukchi Sea. Ecological Applications.

Appendix S5: Estimating unharvested survival probability

Estimates of survival for independent polar bears (i.e., ≥ 2 years old) from the Chukchi Sea (CS) integrated population model (CS-IPM; Regehr et al. 2018) represented total apparent survival, defined as the probability of remaining alive, considering all sources of mortality, and not permanently emigrating from the study area. We adjusted estimates of total survival to exclude human-caused mortality, as follows:

$$\sigma = \sigma^{total}/(1 - H/N), \qquad \text{[eqn S1]}$$

where σ is unharvested survival (Appendix S6, Table S1), σ^{total} is total survival, H is the number of bears removed by humans, and N is abundance. Thus, H/N is the harvest mortality rate. We performed these calculations for polar bears in different life-cycle stages (Figure 2) using stage-specific harvest data (see below) and mean estimates of stage-specific abundance from the CS-IPM. Equation S1 assumed that human-caused mortality is additive within a given year, whereas density-dependent functions in the matrix projection model allowed vital rates to respond in a compensatory manner to changes in density across years. It was necessary to parameterize the projection model using estimates of unharvested survival (i.e., instead of total survival) to evaluate the subpopulation's capacity to grow in the absence of harvest, and to evaluate the effects of harvest levels that differ from the actual harvest during the period 2008–2016.

To estimate unharvested survival using equation S1, harvest data for Alaska were obtained from the U.S. Fish and Wildlife Service (USFWS) Marking, Tagging, and Reporting Program (USFWS, *unpublished data*). Average annual harvest within the U.S. portion of the CS

subpopulation was 23.1 bears/year for the period 2008–2015. To account for likely under-reporting in U.S. harvest (Schliebe et al. 2016) we increased this value by 15% (B. Benter, USFWS, *personal communication*), resulting in 27.2 bears/year as the American contribution to *H*. To estimate the life-cycle stage composition of the harvest we used hunter-reported sex (available for 84% of the harvest sample), hunter-reported age class (available for 90% of the harvest sample), and ages estimated from counting the cementum annuli in vestigial premolar teeth (Calvert and Ramsay 1998) of harvested bears that were submitted by hunters (available for 50% of the harvest sample). For the sample of bears with all three data types, we used the numeric age data to estimate the distribution of life-cycle stages (Figure 2) corresponding to each combination of hunter-reported sex and hunter-reported age class. We then assumed that this distribution applied to bears that had sex and age-class data but did not have numeric age data. Bears without age-class data were assumed to be adults and bears without sex data were assumed to have the same sex distribution as harvested bears of known sex. Polar bear harvest in the U.S. portion of the CS subpopulation is described in detail in Schliebe et al. (2016).

Harvest information for Chukotka, obtained from an interview survey of hunters and community members conducted in 2011–2012 (Kochnev and Zdor 2016), suggested an average annual harvest of approximately 32 bears/year (range 18 – 52 bears/year). For analyses, we represented the average annual harvest in Chukotka for the period 2008–2015 as a uniform distribution Unif(18,52). The mean of this distribution was 35 bears/year, which is approximately 9% higher than the suggested point estimate of 32 bears/year. We considered this reasonable because harvest levels in Kochnev and Zdor (2016) were presented as minimum estimates. We specified the sex composition of independent bears harvested in Chukotka as 43% female and 57% male (A. Kochnev, Russian Academy of Sciences, *personal communication*),

and we assumed that age and reproductive status were like the Alaskan harvest. Although data were not available to evaluate this assumption, it was broadly consistent with qualitative harvest information in Kochnev and Zdor (2016), for example that harvest of adult females with dependent young (i.e., cubs-of-the-year [C0] or yearlings [C1]) is currently uncommon in Chukotka, which also is the case in Alaska (Schliebe et al. 2016).

We estimated empirical harvest rates during the period 2008–2016 based on estimates of harvest level and associated uncertainty described above and estimates of abundance from the CS-IPM. The resulting composition of the harvest, for the purpose of calculating unharvested survival and creating harvest vulnerability vectors for use in the matrix projection model, was approximately 0.12 subadult female (life-cycle stages 1 and 2; Figure 1), 0.19 adult female without dependent young (stages 3 and 4), 0.05 adult female with dependent young (stages 5 and 6), 0.33 subadult male (stages 7-9), and 0.31 adult male (stage 10).

LITERATURE CITED

Kochnev, A., and E. Zdor. 2016. Harvest and Use of Polar Bears in Chukotka: Results of 1999-2012 Studies. Published in partnership with WWF-Russia, Association of Traditional Marine Mammal Hunters of Chukotka, Pacific Fisheries Research Center, and the Institute of Biological Problems of the North, Far East Branch, Russian Academy of Sciences, Moscow, Russia.

Regehr, E. V., N. J. Hostetter, R. R. Wilson, K. D. Rode, M. S. Martin, and S. J. Converse. 2018. Integrated Population Modeling Provides the First Empirical Estimates of Vital Rates and Abundance for Polar Bears in the Chukchi Sea. Sci. Rep. 8:16780.

Schliebe, S., B. Benter, E. V. Regehr, L. Quakenbush, J. Omelak, M. Nelson, and K. Nesvacil. 2016. Co-management of the Alaskan harvest of the Alaska–Chukotka polar bear subpopulation: How to implement a harvest quota. Wildlife Technical Bulletin ADF&G/DWC/WTB-2016-15, Division of Wildlife Conservation, Alaska Department of Fish and Game, Juneau, Alaska.
