# Supplementary material for: Demographic risk assessment for a harvested species threatened by climate change: polar bears in the Chukchi Sea
Source: Ecol Appl. 2021 Oct 26;31(8):e02461. doi: 10.1002/eap.2461 (PMC9286533; doi:10.1002/eap.2461)
Supplement: Supplementary file 6 — Appendix S6 [file EAP-31-0-s004.pdf]

Supporting Information. Regehr, E.V., M.C. Runge, A. Von Duyke, R.R. Wilson, L. Polasek, K.D. Rode, N.J. Hostetter, and S.J. Converse. 2021. Demographic risk assessment for a harvested species threatened by climate change: polar bears in the Chukchi Sea. Ecological Applications.

Appendix S6: Vital rate scenarios

We evaluated harvest risk using two vital rate scenarios that corresponded to different representations of the demographic status of the CS subpopulation. This was relevant because Regehr et al. (2018) indicated that estimates of survival for independent bears from the CS-IPM may have included negative bias due to un-modeled heterogeneity in recapture probabilities and movement probabilities (Peñaloza et al. 2014). This type of bias is a known problem for capture-recapture studies of mobile species when the sampling area is smaller than the subpopulation range, and can lead to biased inference if not accounted for during demographic analyses that rely on estimated vital rates (Regehr et al. 2009).

For both scenarios, prior to analyses we removed vital rate samples corresponding to biologically implausible conditions. Samples were considered implausible if they corresponded to an asymptotic maximum intrinsic growth rate (r_{max}) less than 0 or greater than 0.10. The lower constraint was imposed because it is highly unlikely that conditions experienced by the CS subpopulation during the period 2008–2016 resulted in zero potential for positive growth, which would mean that the subpopulation is guaranteed to go extinct even if environmental conditions remain stable (see below). The upper constraint was imposed because r_{max} = 0.10 is at the upper limit of estimated growth rates for polar bears (Regehr et al. 2017), and near the theoretical upper limit based on species life history. Some vital rate samples did not meet these conditions due to the large amount of process and sampling variation in posterior distributions from the CS-IPM.

Scenario 1

Scenario 1 consisted of estimates of abundance and vital rates directly from the CS-IPM, without adjustment stemming from concerns about negative bias in survival (Table S1). To use the vital rates in the harvest risk assessment, it was necessary to specify the subpopulation size (N) relative to environmental carrying capacity (K) to which the vital rates were referenced (i.e., relative density [N/K]). In practice, it is difficult to estimate relative density because K is usually not known (Gerrodette and Demaster 1990). However, it may be possible to infer relative density based on knowledge of N, the rate of human-caused removals, and species-specific population dynamics. For example, harvest strategies designed to achieve maximum sustainable yield are likely to result in relative densities corresponding to a subpopulation size near maximum net productivity level (*MNPL*), defined as the subpopulation size that results in the greatest net annual increment in numbers resulting from reproduction minus losses due to natural mortality. For most polar bear subpopulations a total harvest rate (i.e., percentage of the total population removed each year) of 4.5% or higher, at a 2:1 male-to-female sex ratio, is required to achieve maximum sustainable yield (Taylor et al. 1987, Regehr et al. 2017). The median total harvest rate for the CS subpopulation during the period 2008–2016 was approximately 2.0%, calculated using estimates of abundance from the CS-IPM and empirical harvest data (Appendix S5). The fact that 2.0% is lower than 4.5% suggests that relative density of the CS subpopulation may have been above *MNPL*, although this cannot be confirmed due to uncertainty in demographic parameters and harvest levels, and an incomplete understanding of density-dependent regulation for polar bears (Derocher and Taylor 1994). To accommodate this uncertainty, for each sample of vital rates under scenario 1 we randomly selected a reference density from a uniform distribution $\mathrm{Unif}(0.50, 0.94)$. The upper limit of 0.94 was established based on exploratory projections using a simple population model with a theta-logistic equation for density

dependence, which USFWS (2016) presented as an approximation of the more complex density-dependent functions in Regehr et al. (2017). Specifically, using this model, 0.94 is the equilibrium density for a hypothetical population with r_{max} = 0.10 (the plausible upper limit used in this report) and a total harvest rate of 2.0%.

Scenario 2

Scenario 2 consisted of vital rates from the CS-IPM, similar to scenario 1, but with survival of independent bears adjusted to result in a mean asymptotic growth rate referenced to a relative density corresponding to *MNPL* (r_{MNPL}) of 0.05, based on the case studies for polar bears reviewed in Regehr et al. (2017). We derived the survival rates for scenario 2 (Table S1) by making an additive adjustment to survival on the logit scale, followed by back-transformation to the probability scale, thus constraining the adjusted estimates to the interval [0,1]. A normal random deviate was included to avoid variance shrinkage. Scenario 2 vital rates were referenced to a relative density corresponding to *MNPL*, to ensure that the capacity for subpopulation growth under scenario 2 was close to empirical estimates for other subpopulations (Regehr et al. 2017).

Scenario 2 represents the assumption that the CS subpopulation was capable of typical growth rates for polar bears during the period 2008–2016. We considered this to be reasonable based on multiple lines of evidence for productivity of the CS subpopulation, including estimates of reproductive parameters from the CS-IPM that are average-to-high for the species (Regehr et al. 2018), indices of recruitment for the CS subpopulation during the period 2008–2016 that were similar to values from the 1980s and 1990s (Rode et al. 2014, 2021; Regehr et al. 2018), indices of positive body condition (Rode et al. 2014, 2021) and low springtime fasting rates (Rode et al.

2017), observations of healthy polar bears in western Alaska from TEK (Braund et al. 2018), and the positive status of ice-dependent seals in the region (Crawford et al. 2015).

LITERATURE CITED

Braund, S. R., P. B. Lawrence, E. G. Sears, R. K. Schraer, B. Adams, T. Hepa, J.C. George, and A.L. Von Duyke. 2018. Polar Bear TEK: A Pilot Study to Inform Polar Bear Management Models. North Slope Borough Department of Wildlife Management Research Report: NSB.DWM.RR.2018-01. Utqiaġvik, Alaska USA.

Crawford, J. A., L. T. Quakenbush, and J. J. Citta. 2015. A comparison of ringed and bearded seal diet, condition and productivity between historical (1975-1984) and recent (2003-2012) periods in the Alaskan Bering and Chukchi seas. Prog. Oceanogr. 136:133-150.

Derocher, A.E. and M.K. Taylor. 1994. Density-dependent population regulation of polar bears. Density-Dependent Population Regulation in Black, Brown, and Polar Bears (ed. M.K. Taylor), pp. 25–30. International Conference on Bear Research and Management Monograph Series No. 3, Washington, DC, USA.

Gerrodette, T., and D. P. Demaster. 1990. Quantitative determinatino of Optimum Sustainable Population level. Mar. Mam. Sci. 6:1-16.

Peñaloza, C. L., W. L. Kendall, and C. A. Langtimm. 2014. Reducing bias in survival under nonrandom temporary emigration. Ecol. Appl. 24:1155-1166.

Regehr, E. V., M. Ben-David, S. C. Amstrup, G. M. Durner, and J. S. Horne. 2009. PhD Dissertation. Chapter 4. Quantifying bias in capture-recapture studies for mobile species: a case study with polar bears; in Polar bear (Ursus maritimus) demography in relation to Arctic sea ice decline. University of Wyoming, Laramie, Wyoming USA.

Regehr, E. V., N. J. Hostetter, R. R. Wilson, K. D. Rode, M. S. Martin, and S. J. Converse. 2018. Integrated Population Modeling Provides the First Empirical Estimates of Vital Rates and Abundance for Polar Bears in the Chukchi Sea. Sci. Rep. 8:16780.

Regehr, E. V., R. R. Wilson, K. D. Rode, M. C. Runge, and H. Stern. 2017. Harvesting wildlife affected by climate change: a modeling and management approach for polar bears. J. Appl. Ecol. 54:1534-1543.

Rode, K. D., E. V. Regehr, J. F. Bromaghin, R. R. Wilson, M. St. Martin, J. A. Crawford, and L. T. Quakenbush. 2021. Seal body condition and atmospheric circulation patterns influence polar bear body condition, recruitment, and feeding ecology in the Chukchi Sea. Global Change Biol. doi: 10.1111/gcb.15572.

Rode, K. D., E. V. Regehr, D. C. Douglas, G. Durner, A. E. Derocher, G. W. Thiemann, and S. M. Budge. 2014. Variation in the response of an Arctic top predator experiencing habitat loss: feeding and reproductive ecology of two polar bear populations. Global Change Biol. 20:76-88.

Rode, K. D., R. R. Wilson, D. C. Douglas, V. Muhlenbruch, T. C. Atwood, E. V. Regehr, E. S. Richardson, N. W. Pilfold, A. E. Derocher, G. M. Durner, I. Stirling, S. C. Amstrup, M. S. Martin, A. M. Pagano, and K. Simac. 2017. Spring fasting behavior in a marine apex predator provides an index of ecosystem productivity. Global Change Biol. 10.1111/gcb.13933:1-14.

Taylor, M. K., D. P. DeMaster, F. L. Bunnell, and R. E. Schweinsburg. 1987. Modeling the sustainable harvest of female polar bears. J. Wildl. Manage. 51:811-820.

USFWS (U.S. Fish and Wildlife Service). 2016. Polar Bear (*Ursus maritimus*) Conservation Management Plan, Final. U.S. Fish and Wildlife, Region 7, Anchorage, Alaska, USA. 104 pp.

TABLES

Table S1. Estimates of unharvested survival for the Chukchi Sea polar bear subpopulation. Estimates were derived by adjusting estimates of total survival from Regehr et al. (2018) using estimates of harvest mortality rate (Appendix S5). Survival probability σ_i is the annual probability of survival of an individual in stage *i*, as defined in the life-cycle graph underlying the matrix projection model (Figure 2). Scenario 1 is based directly on estimates from Regehr et al. (2018) whereas survival rates under scenario 2 were adjusted upwards to account for potential bias. Values are reported as the mode and 95% credible intervals (CRI) for consistency with Regehr et al. (2018).

	scenario 1			scenario 2		
	mode	CRI_{lower}	CRI_{upper}	mode	CRI_{lower}	CRI_{upper}
σ_1	0.82	0.70	0.91	0.88	0.75	0.94
σ_2	0.82	0.70	0.91	0.88	0.75	0.94
σ_3	0.91	0.88	0.95	0.94	0.90	0.97
σ_4	0.91	0.88	0.95	0.94	0.90	0.97
σ_5	0.91	0.88	0.95	0.94	0.90	0.97
σ_6	0.91	0.88	0.95	0.94	0.90	0.97
σ_7	0.75	0.63	0.86	0.83	0.69	0.92
σ_8	0.75	0.63	0.86	0.83	0.69	0.92
σ_9	0.75	0.63	0.86	0.83	0.69	0.92
σ_{10}	0.91	0.85	0.96	0.94	0.88	0.98
